# Multi-objective optimization for EEG channel selection and accurate intruder detection in an EEG-based subject identification system

**DOI:** 10.1038/s41598-020-62712-6

**Published:** 2020-04-03

**Authors:** Luis Alfredo Moctezuma, Marta Molinas

**Affiliations:** Department of Engineering Cybernetics, Norwegian University of Science and Technology, 7491 Trondheim, Norway

**Keywords:** Biomedical engineering, Applied mathematics, Computational neuroscience, Biosensors, Computational models, Computational neuroscience, Data processing, Machine learning, Biomarkers

## Abstract

We present a four-objective optimization method for optimal electroencephalographic (EEG) channel selection to provide access to subjects with permission in a system by detecting intruders and identifying the subject. Each instance was represented by four features computed from two sub-bands, extracted using empirical mode decomposition (EMD) for each channel, and the feature vectors were used as input for one-class/multi-class support vector machines (SVMs). We tested the method on data from the event-related potentials (ERPs) of 26 subjects and 56 channels. The optimization process was performed by the non-dominated sorting genetic algorithm (NSGA), which found a three-channel combination that achieved an accuracy of 0.83, with both a true acceptance rate (TAR) and a true rejection rate (TRR) of 1.00. In the best case, we obtained an accuracy of up to 0.98 for subject identification with a TAR of 0.95 and a TRR 0.93, all using seven EEG channels found by NSGA-III in a subset of subjects manually created. The findings were also validated using 10 different subdivisions of subjects randomly created, obtaining up to 0.97 ± 0.02 of accuracy, a TAR of 0.81 ± 0.12 and TRR of 0.85 ± 0.10 using eight channels found by NSGA-III. These results support further studies on larger datasets for potential applications of EEG in identification and authentication systems.

## Introduction

An authentication system includes a stage in which the data is used in a multi-class model with all the subjects in the dataset to identify a specific subject. It also includes a verification step to compare the data from the claimed subject with that of the true subject alone in the dataset to detect whether the subject is an intruder or not. The order of these stages may differ depending on the approach.

Subject identification and authentication based on electroencephalography (EEG) has been presented as a potential candidate for the creation of new biometric systems. However, certain aspects need to be analyzed and improved before reaching an industrial level application. One is intruder detection, which is an essential security layer in any secure system. This is also important for the development of portable low-density EEG devices that retain similar accuracy as high-density EEG. Here, we consider both problems, authentication, and verification.

A number of different paradigms are used to stimulate and record EEG signals in various subject identification approaches: imagined speech^1–3^, resting-state potentials^4,5^, and event-related potentials (ERPs)^6^. ERPs are very low-voltage signals emitted by the human brain that appears on the scalp in response to specific events or stimuli. They produce several well-known patterns, of which one of the most studied is the P300 peak, which occurs approximately 300 ms after the onset of the stimulus^7,8^.

In general, resting-state potentials and ERPs have been shown to be good candidates for a new biometric system for which there are several different state-of-the-art approaches^6,9–12^, with the localization of the relevant channels differing, depending on the paradigm. Here, we used the ERP approach to stimulate the EEG signals.

Four main objectives need to be considered to ensure an optimal global configuration: 1) select the minimum number of relevant EEG channels, 2) reject all intruders, 3) accept all subjects that are already in the system, and 4) identify the subject by multi-class classification. Consideration of these four objectives ensures high-quality results.

In addition to classification accuracy, the evaluation of biometric systems may also include consideration of the true acceptance rate (TAR), false acceptance rate (FAR), and true rejection rate (TRR). The TAR is the percentage of times a system correctly verifies a true claim of identity, FAR the percentage of intruders that are verified as non-intruders, and TRR the percentage of times it correctly rejects an intruder.

An approach with one-second EEG signals from the FP1 and FP2 channels and a 256-Hz sample rate during the resting state has been proposed for a biometric system, extracting features directly from the raw data and using Fisher’s discriminant analysis^10^, obtaining an up to 0.966 TAR and a 0.034 FAR. Another approach used two-second EEG signals from the FP1 and FP2 channels, with a 2048-Hz sample rate, and authors used a set of classifiers to perform multi-class classification^11^. They obtained an accuracy of 0.93 and a false positive identification rate of 0.165. A third approach presented the results of a study using the Cz EEG channel, which was selected manually, on 20 subjects during the resting-state^12^, obtaining a TAR of 1.0 and TRR of over 0.8. None of these studies attempted to systematically select the minimal number of optimal channels to perform the task.

An important element is dimensionality reduction, which can be tackled through channel selection and feature extraction. Several approaches can be used to accomplish this task, including those based on methods such as principal component analysis (PCA)^13^, discrete wavelet transform (DWT)^3^, empirical mode decomposition (EMD)^2,3,6^, and even approaches using raw data as input for different configurations of neural networks (NN)^14,15^. Based on the current state-of-the-art and the results of our previous studies, we used EMD for sub-band extraction and then extracted four features for each: instantaneous and Teager energy and Higuchi and Petrosian fractal dimensions.

Here, we used a one-class support vector machine (SVM) classifier with a radial basis function (RBF) kernel to create an intruder detection layer. Then, we used the linear SVM classifier for multi-class classification, which was evaluated for accuracy by 10-fold cross-validation. Considering a common configuration for all tasks, one-class classification, multi-class classification, and channel selection presents an optimization problem that can limit the results and be computationally expensive. We tested performing searches in the limited space using the non-dominated sorting genetic algorithm (NSGA), which uses the Pareto-optimal solutions and a niche method to maintain stable sub-populations of good points^16^. The NSGA-II and NSGA-III methods were tested and the results compared, given their use of the elitism approach and predefined reference points.

## Results

### General configuration for intruder detection using all EEG channels

**Figure 1:**
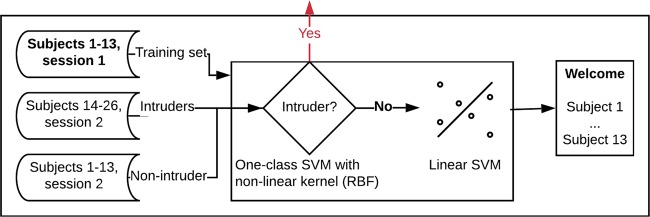
Flowchart of the first approach for intruder detection and subject identification.

As described below, we used a two-stage approach for the entire process, illustrated in Fig. 1. Briefly, we created a one-class SVM model in which we aimed to train the model to recognize subjects that are already in the system and to reject those who are not (Intruders). In the first part of this experiment, we thus trained the model using subjects with IDs 1–13 (non-intruder) and only EEG signals from session one, using 30 instances and all EEG channels (56 channels). We then used the EEG signals from all the subjects of session two, considering subjects 14–26 as intruders, to validate the model (see Fig. 1). We evaluated the results using the TAR, TRR, and accuracy of multi-class classification (Table 1).

**Table 1:** TAR, TRR, and accuracy for subject identification and authentication with EEG data from all channels using different **nu** and **gamma** values for one-class SVM.

	Subjects	*nu*	*gamma*	TAR	TRR	Accuracy
Non-intruders	1–13	0.01	0.01	**0.923**	—	0.98 ± 0.2
Intruders	14–26			—	**0.083**	—
Non-intruders	1–13	0.10	0.10	**0.545**	—	
Intruders	14–26			—	**0.449**	—
Non-intruders	14–26	0.01	0.01	**0.951**	—	1.00 ± 0.0
Intruders	1–13			—	**0.212**	—
Non-intruders	14–26	0.10	0.10	**0.495**	—	
Intruders	1–13			—	**0.551**	—

Table 1 presents an example of the results using subjects 1–13 as non-intruders and subjects 14–26 as intruders. They show that approximately 90% of the subjects were correctly accepted, but that also that only approximately 8% of the intruders were correctly rejected. However, changing the *nu* and *gamma* parameters for the SVM RBF changed the TAR and TRR to approximately 50% in both cases.

Given that all subjects with access (subjects 1–13) passed the first layer, we created a multi-class classifier for subject identification. We experimentally defined and used a SVM with a linear kernel to create this model, because of the results obtained in previous studies and also because it was found to be the best solution experimentally. The flowchart of the complete method is presented in Fig. 1. The accuracy we obtained following 10-fold cross-validation was **0.98**, with a standard deviation of **0.02** (Table 1).

We used this approach because our aim was to find the best configuration for the entire process. Creating a model using only the subjects with correct permission who passed the first layer would have affected the results and therefore not be valid.

### Solving the four-objective optimization problem using NSGA-II with subjects 1–13 as non-intruders and 14–26 as intruders

From here on, we present experiments that simultaneously considered all the problems to investigate whether there is a particular combination that can solve them. The experiment consisted of solving the optimization problem defined in section *methods* using NSGA-II. It consists of finding the best *nu* and *gamma* for the SVM with the RBF kernel to increase the TAR, TRR, and accuracy of subject identification or maintain them as high as possible for previous configurations, while using the smallest number of possible EEG channels. In short, we performed NSGA-II for the channel selection method using the first 56 genes in a chromosome to represent the EEG channels and then four genes each to select the best *nu* and *gamma* parameters, obtaining thus a chromosome of 64 genes.

**Figure 2:**
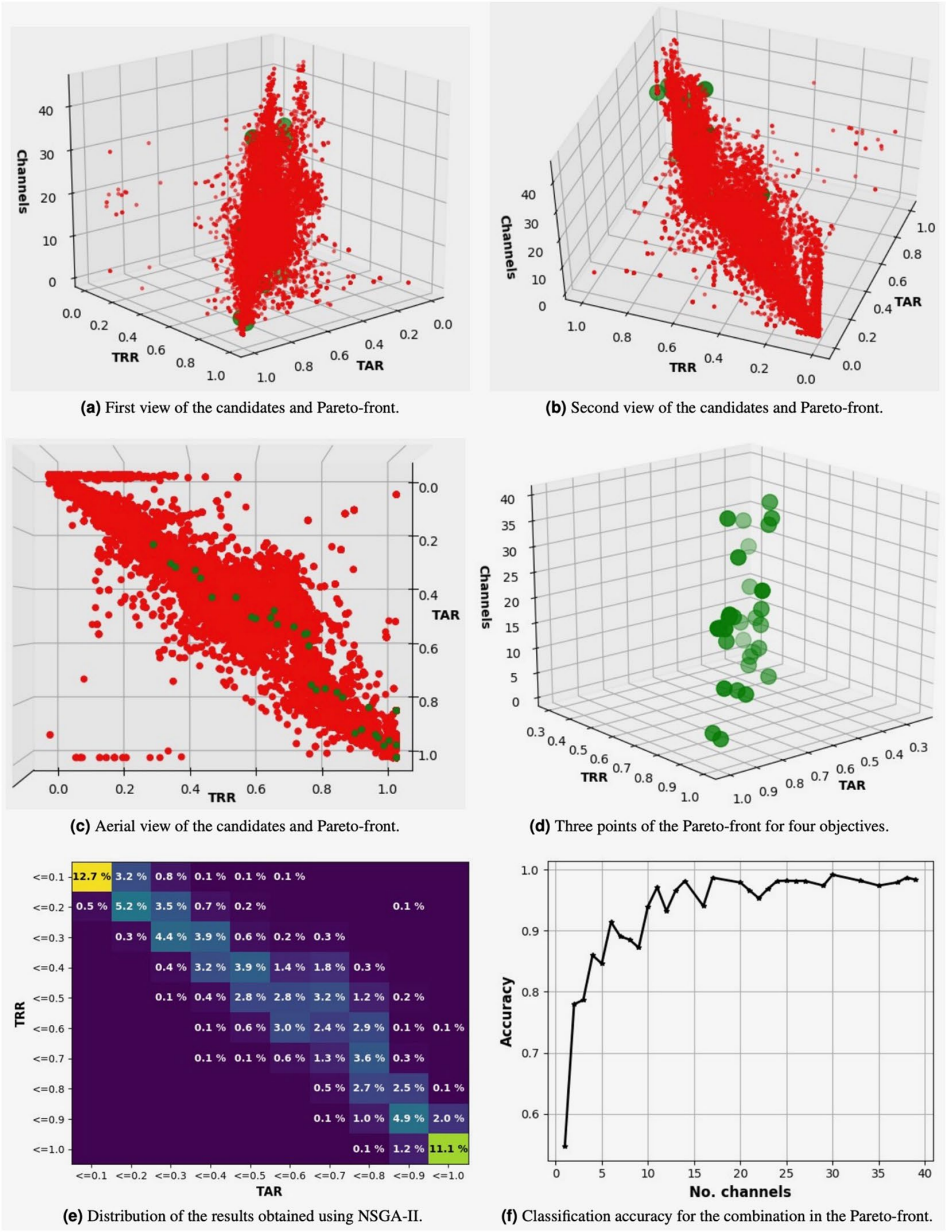
Four different views of the results obtained using subjects 1–13 and intruders 14–26. The Pareto-front is presented as green points and the candidates as red points (Sub-figs. 2a–d). Distribution of the results obtained and the evolution of the classification accuracy (Sub-figs. 2e,f).

Several plots of the results obtained considering the four objectives are presented in Fig. 2 to illustrate the importance of the optimization process, as only 11.11% of the possible channel combinations resulted in a TAR and TRR between 0.9 and 1.0 (Sub-fig. 2e). The classification accuracy in relationship to the number of channels used and the Pareto-front are shown in Sub-fig. 2d,f.

The results for the Pareto-front for all objectives are presented in Table 2. NSGA-II found a two-channel combination for which we obtained a TAR of 0.91, TRR of 0.88, and an accuracy of 0.78 for subject identification. NSGA-II also found a 12-channel combination for which the accuracy of subject identification was 0.93, the TAR 0.93, and the TRR 0.95. This result shows that it is possible to reduce the number of channels from 23, 24, etc (which gave similar accuracies) to almost half using this approach.

**Table 2:** TAR, TRR, and accuracies obtained in the Pareto-front for four objectives solved with NSGA-II using subjects 1–13 as non-intruders.

No. channels	Accuracy	TAR	TRR	*nu*	*gamma*
1	0.55	0.90	0.90		
2	**0.78**	**0.91**	**0.88**	**0.0001**	**0.9**
3	0.79	0.34	0.42		
4	0.86	0.31	0.35		
5	0.85	0.50	0.58		
6	0.91	0.56	0.74		
7	0.89	0.51	0.60		
**8**	**0.89**	**0.79**	**0.85**	**0.0010**	**0.9**
**9**	**0.87**	**0.82**	**0.92**	**0.0001**	**0.2**
10	0.94	0.53	0.66		
11	0.97	0.43	0.47		
**12**	**0.93**	**0.93**	**0.95**	**0.0001**	**0.9**
13	0.97	0.43	0.54		
14	0.98	0.51	0.64		
16	0.94	0.76	0.77		
17	0.99	0.37	0.44		
20	0.98	0.61	0.75		
21	0.97	0.76	0.80		
22	0.95	0.25	0.30		
23	0.97	0.92	0.94		
24	0.98	0.96	0.96		
25	0.98	1.00	1.00		
26	0.98	0.94	0.98		
27	0.98	0.96	1.00		
29	0.97	0.93	0.96		
30	0.99	0.83	1.00		

### Solving the four-objective optimization problem using NSGA-II with subjects 14–26 as non-intruders and subjects 1–13 as intruders

With the aim of providing a more global result, we repeated the previous experiment using the same configuration but now considering subjects 14–26 as non-intruders and subjects 1–13 as intruders. The results obtained for the four objectives are presented in Table 3.

**Table 3:** TAR, TRR, and accuracies obtained for the first 30 EEG channels in the Pareto-front for four objectives solved with NSGA-II using subjects 14–26 as non-intruders.

No. channels	Accuracy	TAR	TRR	*nu*	*gamma*
1	0.53	0.70	0.70		
2	0.62	0.31	0.31		
**3**	**0.83**	**1.00**	**1.00**	**0.00001**	**0.6**
4	0.87	0.41	0.37		
5	0.88	0.49	0.49		
6	0.96	0.81	0.73		
7	0.96	0.74	0.78		
**8**	**0.91**	**0.88**	**0.89**	**0.3000**	**0.8**
9	0.97	0.52	0.54		
**10**	**0.97**	**0.90**	**0.91**	**0.0005**	**0.6**
11	0.96	0.83	0.88		
12	0.97	0.55	0.56		
13	0.98	0.40	0.52		
14	0.98	0.80	0.84		
15	0.98	0.50	0.56		
**16**	**1.00**	**1.00**	**1.00**	**0.00001**	**0.6**
17	0.99	0.73	0.65		
18	0.98	0.93	0.93		
19	0.99	0.38	0.59		
20	0.99	0.47	0.57		
21	0.98	0.74	0.71		
22	0.99	0.99	0.99		
23	0.98	0.76	0.72		
24	1.00	0.74	0.64		
25	1.00	0.99	0.99		
26	1.00	1.00	0.99		
27	1.00	1.00	1.00		
28	1.00	0.96	0.96		
29	1.00	0.95	0.97		
30	1.00	1.00	1.00		

As in the previous experiment, we obtained an accuracy of up to 0.83 for subject identification, with both a TAR and TRR of 1.00, using just a three-channel combination (see Table 3). Increasing the classification accuracy for subject identification, while maintaining the same TAR and TRR, required 16 EEG channels, in contrast to the previous experiment for which the optimal number of EEG channels was 12.

Table 3presents the results obtained in the Pareto-front for the first 30 EEG channels, indicating the accuracies obtained, TAR and TRR as well as the *nu* and *gamma* values used for creating the one-class classifiers for obtaining thus TAR and TRR results. We marked in gray the most relevant accuracies, TAR and TRR and the corresponding no. channels used, for those results we also added the *nu* and *gamma* values used in each case, in order to observe if there exist similarities between those cases.

**Figure 3:**
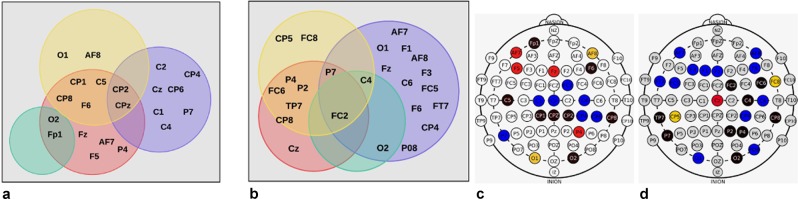
Relevant EEG channel subsets in the Pareto-front for four objectives using NSGA-II, considering subjects 14–26 as intruders in the previous experiment and subjects 1–13 as intruders in the current experiment.

The channel combinations for this and the previous experiments were independent and likely differed. We generated Venn diagrams to compare the channels in the Pareto-front between this and the previous experiment to detect a possible pattern or a more relevant area (Fig. 3). The EEG channels used to obtain the results marked in gray in Table 2 and the channel localization in Sub-fig. 3c are presented in Sub-fig. 3a. The results marked in gray in Table 3 are shown in Sub-fig. 3b and EEG channel localization in Sub-fig. 3d.

Figure 3 shows some channels in a black circle if that channel is in an intersection with one or more subsets. For instance, sub-fig. 3c shows *CPZ* channel in a black circle which mean that it was in one or more subsets, as it is shown in sub-fig. 3a. It is important to highlight these for the discussion of the results and for comparison purposes with the following experiments in this document.

### NSGA-III for solving the four-objective optimization problem

We repeated the previous two experiments for solving the four-objective optimization problem with the same configuration, but now using NSGA-III. A comparison between the results obtained in the Pareto-front in the two experiments, using subjects 1–13 for the training (subjects 1–13 as non-intruders and 14–26 as intruders) and subjects 14–26 for training (subjects 14–26 as non-intruders and 1–13 as intruders), is shown in Table 4.

**Table 4:** TAR, TRR, and accuracies obtained in the Pareto-front when using 7–15 EEG channels with four objectives solved with NSGA-III using subjects 1–13 as non-intrudes and 14–26 as non-intruders and vice-versa.

Subjects	Eval.	No. channels								
		7	8	9	10	11	12	13	14	15
1–13	**Accuracy**	0.96	0.96	**0.98**	**0.98**	0.98	0.99	0.99	**0.99**	0.98
	**TAR**	0.41	0.41	**0.94**	**0.94**	0.61	0.70	0.60	**1.00**	0.29
	**TRR**	0.47	0.48	**0.94**	**0.94**	0.84	0.85	0.60	**1.00**	0.37
	***nu***			**0.0005**	**0.0001**				**0.0005**	
	***gamma***			**0.1**	**0.1**				**0.1**	
14–26	**Accuracy**	**0.98**	0.97	0.98	0.97	**0.99**	0.98	1.00	**1.00**	0.99
	**TAR**	**0.95**	0.93	0.90	0.93	**0.95**	0.94	0.93	**0.94**	0.72
	**TRR**	**0.93**	0.93	0.91	0.94	**0.95**	0.92	0.93	**0.95**	0.83
	***nu***	**0.0100**				**0.0001**			**0.0001**	
	***gamma***	**0.7**				**0.9**			**0.9**	

In this experiment, we found subsets with 9, 10, and 14 optimal EEG channels using subjects 1–13 as non-intruders and subsets with 7, 11, and 14 EEG channels using subjects 14–26 as non-intruders. As in the previous experiments, a comparison of several relevant subsets presented in Table 4 is presented in Fig. 4 for both cases, either using subjects 1–13 as non-intruders (Sub-figs. 4a,c) or 14–26 as non-intruders (Sub-figs. 4b,d).

**Figure 4:**
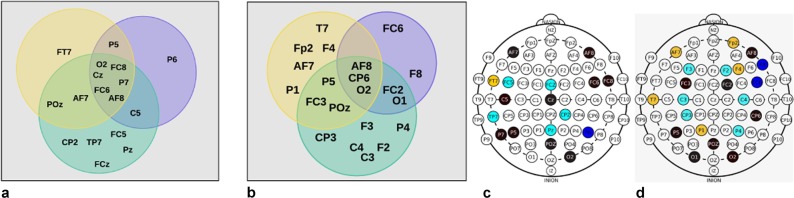
Relevant EEG channel subsets in the Pareto-front for four objectives using NSGA-III, considering subjects 14–26 as intruders in the previous experiment and subjects 1–13 as intruders in current experiment.

Figure 4 presents a comparison between different subsets found by NSGA-III when using Subjects 1–13 as non-intruders and also using subjects 1–13 as intruders. This fig. shows a lower number of channels in the interceptions, but it also shows that most of the EEG channels used for obtaining the best results presented in Table 4 were obtained using channels around the parietal and occipital areas.

### Testing the proposal in 10 random subdivisions of subjects using NSGA-II and NSGA-III

In previous experiments, we presented the results obtained using different subsets manually selected with 50% of the subjects as non-intruders and 50% as intruders (I.e subjects 1–13 as non-intruders and 14–26 as intruders, and vice-versa.). We also presented the differences found when using NSGA-II or NSGA-III. However to provide a more general validation of our proposal, we created random subsets with 50% of the subjects as non-intruders and 50% as intruders, then we solved the optimization problem by considering the four objectives at the same time. This process is repeated 10 times, obtaining thus, 10-fold cross-validation of the proposed method. We also repeated the experiment using both algorithms, NSGA-II and NSGA-III, the mean results and the standard deviation are presented in Table 5.

**Table 5:** Mean TAR, TRR, and accuracies obtained in the Pareto-front when using 7–15 EEG channels validated in 10 random subdivisions of all the subjects, using 50% as intruders and 50% as non-intruder.

Method	Eval.	No. channels								
		7	8	9	10	11	12	13	14	15
NSGA-II	**Acc**.	0.96 ± 0.02	0.96 ± 0.01	0.97 ± 0.02	0.98 ± 0.02	1.00 ± 0.00	0.99 ± 0.01	1.00 ± 0.00	1.00 ± 0.00	0.99 ± 0.01
	**TAR**	0.74 ± 0.18	0.81 ± 0.18	0.59 ± 0.07	0.74 ± 0.05	0.81 ± 0.08	0.61 ± 0.25	0.81 ± 0.17	0.86 ± 0.13	0.90 ± 0.10
	**TRR**	0.85 ± 0.14	0.79 ± 0.10	0.68 ± 0.16	0.87 ± 0.13	0.69 ± 0.18	0.89 ± 0.10	0.88 ± 0.12	0.90 ± 0.09	0.94 ± 0.06
NSGA-III	**Acc**.	0.97 ± 0.03	0.97 ± 0.01	0.97 ± 0.02	0.98 ± 0.02	1.00 ± 0.00	1.00 ± 0.00	1.00 ± 0.00	1.00 ± 0.00	1.00 ± 0.00
	**TAR**	0.72 ± 0.14	0.81 ± 0.12	0.64 ± 0.14	0.79 ± 0.07	0.86 ± 0.08	0.78 ± 0.15	0.82 ± 0.17	0.86 ± 0.13	0.92 ± 0.08
	**TRR**	0.74 ± 0.12	0.85 ± 0.10	0.65 ± 0.21	0.85 ± 0.13	0.80 ± 0.13	0.89 ± 0.10	0.89 ± 0.10	0.89 ± 0.09	0.94 ± 0.02

The results presented in Table 5, shows that when considering 10 random partitions of the subjects as non-intruders or intruder, the mean accuracy decreases in both cases when using NSGA-II and NSGA-III. Also the standard deviation when using a lower number of channels than 10, is higher than 10%. This is because in each partition randomly created, the number of the best channel array as well as the best channels, are not the same. For instance, in previous experiment presented in Table 4 it was clearly presented than using subjects 1–13 as non-intruders the best results are obtained with nine EEG channels, but considering subjects 14–26, the best results are obtained using seven channels.

As an example, Table 5 shows that when using eight EEG channels the accuracies, TAR and TRR are similar in both cases, using NSGA-II and NSGA-III. However, the standard deviation is higher than 10% for TAR and TRR, which means that for some subsets of subjects, the best results were not obtained with eight channels, i.e sometimes with seven and sometimes with nine channels as in the previous experiments. In summary, this new experiment has shown that the accuracy for subject identification is consistently high (I.e higher than 0.96 in all the cases, as in the previous experiments exposed), but depending on which subset of subjects is used as intruders or non-intruders, TAR and TRR may vary.

## Discussion

This study shows the promise of a new biometric system using a novel data source. Here, we present an EEG-based biometric system as a good candidate for use in authentication systems. In our previous works, we have studied and compared various paradigms, i.e. resting-state potentials and ERPs, using various types of electrodes, a various number of channels, and channel localization^2–4,6^. Several parameters are yet to be optimized. There are thus no currently available industrial-level EEG-based biometric systems. In the context of designing a portable EEG headset, applications for multi-task purposes and scenarios are being widely studied. We propose using NSGA-based algorithms for the optimization process, with the final objective of reducing the necessary number of EEG channels for subject identification. These algorithms depend upon several parameters, which influence the performance and results. Additionally, these machine-learning algorithms also require the definition of several parameters, which we defined using eight genes of a created chromosome.

Here, we introduced a new scheme for subject identification and authentication, showing that we can identify subjects by their EEG brain signals and distinguish between subjects who were part of the trained dataset from those that are intruders. Using NSGA-II in our first experiments, we found channel subset combinations consisting of only two EEG channels with which we obtained an accuracy of 0.78, with a TAR of 0.91 and a TRR of 0.88. However, eight, nine, or twelve channels were required to increase the value of the results for the objectives when they were applied simultaneously. NSGA-III found subsets with seven, nine, ten, or eleven EEG channels with an accuracy of up to 0.99 and both a TAR and TRR of 1.00. Initially, we aimed at creating a new fixed headset with a limited number of EEG channels, but as the results of this work shows, it is not possible to argue that certain “good” subset works better than other, since there exist different factors that are critical when choosing whether it is better to use a lower number of EEG channels or propose improvements in the classification stage. Our proposal has shown that there exist different channel subsets with which we can obtain high accuracies, TAR and TRR. However, deeper analysis and more experiments are required to perform within a larger population.

P300 from ERPs have shown to be good candidates but they are not the gold standard for this application since there is not yet sufficient research evidence to support it. They have been proposed in this work as candidates since it has shown to exhibit strong signatures unique to the subject and the process does not require any training, which will be essential in a real-life application. In a real scenario, the biometric system can display something on a screen (an image, a weak flashlight beam directly to the eyes, etc.), record the brain activity corresponding to the response to the presentation, and use it for the identification and authentication process. The internal state of the subject, such as the resting state, could also be used as an alternative obtaining specific information on the subject, as discussed in our previous investigation^4^. The EEG channel selection process is in itself interesting because it can provide information about the most relevant areas in the brain for a certain neural task, for a certain subject or group of subjects. This can be analyzed using *a-priori* information related to the paradigm, which can limit the search space and therefore the results.

The results presented in the first experiments show that most of the common channels in the subsets providing the highest accuracy, TAR, and TRR, come from occipital and parietal areas, but there were also some important channels in the frontal area (FC2, FC3, FC6, FC8, F6, AF7, AF8, and Fp1). In short, a final conclusion about the minimum number of necessary EEG channels for Subject identification taking into account the classification accuracy, TAR, and TRR, cannot be proposed solely based on the results of this work since depending on different factors (I.e number of subjects, trials, sessions, feature extraction method, channel selection approach and their parameters, etc.), the minimum necessary number of channels will be different. Additionally, the channel localization for the subsets found differ between them and also they differ if we use NSGA-II or NSGA-III methods, which is clearly presented in Figs. 3 and 4. When considering 10 random subdivisions of the subjects, the mean TAR and TRR decrease, and the standard deviation increases, additionally the *nu* and *gamma* values used are different in each subdivision, but the classification accuracies are maintained similar to our first presented experiments.

The complexity of the analysis can be as high as that required. In our first experiments, we trained a model with EEG signals from session 1 and the authentication and verification process was constructed using EEG signals from session 2. However, an analysis of different sessions from different days/weeks/months is also necessary before a proof of concept, due to brain plasticity and how it can affect this biometric approach. Another important aspect that requires further study is the scalability, to verify the number of subjects that can be added to this system while maintaining similar performance to that when using a small number of subjects. Here, we created a first-layer using the EEG data from all the subjects to search for a method to increase the TAR and TRR. However, we will compare this approach against a model for each subject, with a possible combination of both, to obtain better results for the TAR and TRR. We will focus on all these relevant aspects in future studies involving the optimization of multiple parameters related to the feature extraction and machine learning methods by using discrete values for representing the chromosomes and not only as a binary sequence. Another important aspect to be further investigated is the use of larger datasets with a *k* − *fold* validation, and verify if a possible modification to our proposal can find a single array of best EEG channels subset for different subjects subdivisions randomly created while solving consistently all the defined objectives and the necessary parameters by optimization as in our experiments presented and discussed in this document.

Our research has been focused towards a portable (non-invasive) wireless dry single-channel or low-density EEG system for different applications, which can help the subjects identification process by providing EEG information from different channel combinations by using a movable sensor^4^. Following the results found in this work and other applications proposed previously, we will analyze the possibility of a fixed or movable electrode version of a new EEG headset which can incorporate the best results obtained in this work for subject identification and authentication.

## Methods

Although a research-grade EEG device guarantees a controlled environment and high-quality multi-channel recording, this is offset by the high computational cost, high density, non-portability of the equipment, and the use of inconvenient conductive gels. Depending on the paradigm and the task, certain EEG channels provide only redundant or poor information.

Recent technology development in dry EEG sensors has created new possibilities for the development of new types of portable EEG systems. An important step towards this goal is a reduction in the number of EEG channels while increasing or at least maintaining the same performance as high-density EEG by using multi-objective optimization algorithms.

### Dataset

**Figure 5:**
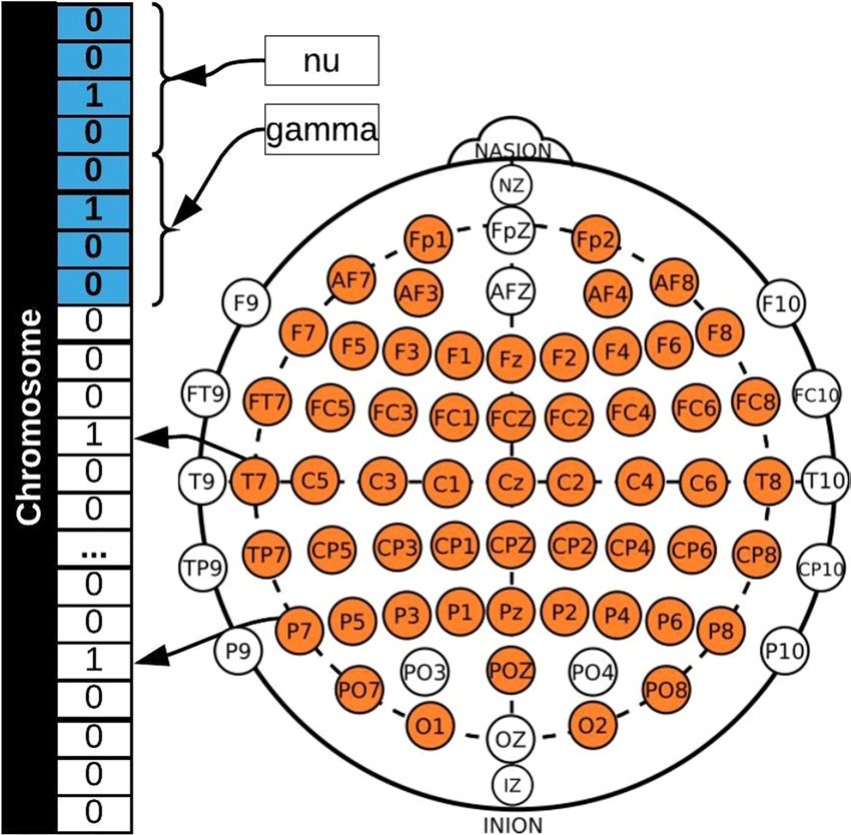
Chromosome created for the NSGA using 56 genes for the EEG channels and eight for the *nu* and *gamma* parameters.

We used EEG signals from 26 subjects (24 right-handed and 2 left-handed), with an average age of 29.2 ± 5.5 years, from 56 passive Ag/AgCl EEG electrodes (VSM-CTF compatible system) which were placed following the extended 10–20 international system (illustrated in Fig. 5). The EEG signals were all referenced to the nose and the ground electrode was placed on the shoulder, the impedances were kept below 10 k*Ω*. The EEG data was collected in five sessions and 60 instances per each session with a sample rate of 600Hz but they were down-sampled at 200 Hz^17^.

**Figure 6:**
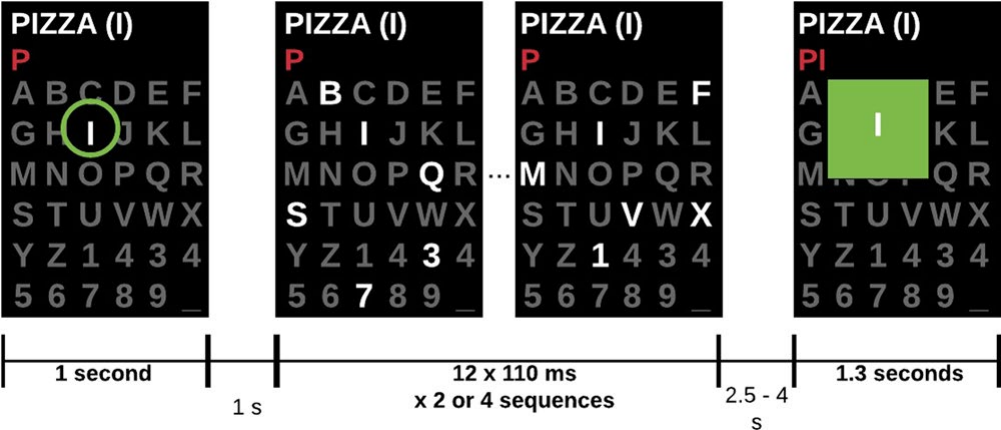
Protocol design for recording positive or negative feedback-related responses^17^.

The protocol used to record the EEG signals used the P300-speller paradigm (as is illustrated in Fig. 6) and introduced in^17^. Briefly, the target letter (the letter to be presented) is indicated by a green circle for 1 s. Then, letters and numbers (6 X 6 items, 36 possible items displayed on a matrix) are flashed in groups of six characters. Next, the display remains blank for a period of resting-state from 2.5 to 4 s. During this random period, the subjects are requested to remember the letter displayed. Then, the letter chosen by the implemented P300 classifier is displayed for 1.3 s. If the presented letter is the one that was previously presented, the subject sends a positive response, otherwise the subject sends a negative response.

An example of a positive feedback-related response corresponding to the target letter *i* is shown in Fig. 6. For the experiments presented here, we used only the positive-feedback responses, therefore, the number of positive-feedback trials can be different between subjects and sessions, and we selected the minimum number of positive-feedback related responses, which is 25 instances per session and per subject.

### Pre-processing

The common average reference (CAR) method was used to improve the signal-to-noise ratio from the EEG signal by removing the common information from all electrodes that was simultaneously recorded. CAR can be computed for an EEG channel ${V}_{i}^{CAR}$, where *i* is the number of the channel, as follows:

1 $${V}_{i}^{CAR}={V}_{i}^{ER}-\frac{1}{n}{\sum }_{j=1}^{n}{V}_{j}^{ER}$$

where ${V}_{i}^{ER}$ is the potential between the *i*-th electrode and the reference, and *n* the number of electrodes.

### Feature extraction

We used EMD to decompose the EEG signals into a set of intrinsic mode functions (IMFs)^18^. Certain redundant IMFs with shape and frequency content different from those of the original signal may appear during the sifting process. These signals show maximum Minkowski distances with respect to the original signal^19^.

We used the closest two IMFs, based on the Minkowski distance, and each IMF was characterized by extracting a set of four features^2^: *Instantaneous energy*, which provides the energy distribution^20^, *Teager energy*, which reflects variations in both the amplitude and frequency of the signal^20,21^, *Higuchi fractal dimension*, which approximates the mean length of the curve using segments of *k* samples and estimates the dimension of a time-varying signal directly in the time domain^22^, and *Petrosian fractal dimension* to provide rapid computation of the fractal dimension of an EEG signal by translating the time series into a binary sequence^23^.

The process for extracting four features for each selected IMF returns eight features per channel and it is repeated for each channel used to then concatenate them to obtain a unique feature vector that represents the EEG signal for each instance.

### Classification

We used a two-layer classification system, which is briefly introduced below.

#### One-class SVM with RBF

The SVM consisted of an unsupervised algorithm that learns a decision function for outlier detection, classifying new data as similar to or different from that of the training set. The radial basis function (RBF) was used as the kernel and certain important parameters required fitting. We found *nu* and *gamma* to be two important basic parameters for our proposed system. The *nu* parameters were an upper bound on the fraction of training errors and a lower bound of the fraction of support vectors that should be in the interval [0, 1]. *Gamma* defines how much influence a single training example has. The larger the *gamma*, the closer other examples must be to be affected and the interval must be greater than 0; normally it is 1∕*no*_*features*.

A grid search can be used to adjust SVM parameters by cross-validation, which has been shown to be powerful and able to significantly improve accuracy. However, it is a very slow process^24^. These parameters differ depending on the size of the feature vector and it is necessary to re-compute them each time. In an optimization problem, this process must be executed multiple times and it is computationally expensive. As explained below, We used eight genes of a bigger chromosome to fit these parameters (four for *nu* and four for *gamma*) using a genetic algorithm (GA).

#### Multi-class linear SVM

We tested a multi-class SVM, which provides a global solution. The classification complexity does not depend on the dimensionality of the feature space and the sensitivity to the number of features is relatively low^25^, as the necessary time to create a model is ${\mathscr{O}}({N}^{3})$, where *N* is the length of the feature vector, and ${\mathscr{O}}(1)+{\mathscr{O}}(N)$ is required to predict the class of a new instance using the created model^26^.

We used accuracy as the metric to evaluate the performance of the process using 10-fold cross-validation.

### EEG channel selection

The EEG channel-selection process is critical for the development of a portable low-cost headset and makes it possible to focus on the EEG channels that contain the most information.

We used a genetic algorithm, as they are used to solve complex optimization and exhaustive search problems^27^. We attempted to solve the multi-objective optimization problem using the non-dominated sorting genetic algorithms (NSGA)^16^, which uses a non-dominated sorting ranking selection method to emphasize good candidates and a niche method to maintain stable sub-populations of good points (Pareto-front).

NSGA-II solved certain problems related to the computational complexity, non-elitist approach, and the need to specify a sharing parameter to ensure diversity in a population presented in the first version. NSGA-II reduced the computational cost from *O*(*MN*^3^) to *O*(*MN*^2^), where *M* is the number of objectives and *N* the population size. Additionally, the elitist approach was introduced by comparing the current population with the previously found best non-dominated solutions^28^.

Later, NSGA-III was introduced, which follows the NSGA-II framework, but uses a set of supplied or predefined reference points that emphasizes population members that are non-dominated, yet close to the supplied set (^29,30^). The predefined set of reference points are used to ensure diversity in the obtained solutions. We used a systematic approach that places points on a normalized hyper-plane that is equally inclined to all objective axes and has an intersection with each axis. For example, in a three-objective optimization problem, the reference points are created on a triangle with apexes at (1, 0, 0), (0, 1, 0), and (0, 0, 1)^30,31^. NSGA-III has shown its efficiency in solving two- to 15-objective optimization problems (^29^).

### Definition of the problem to optimize

**Figure 7:**
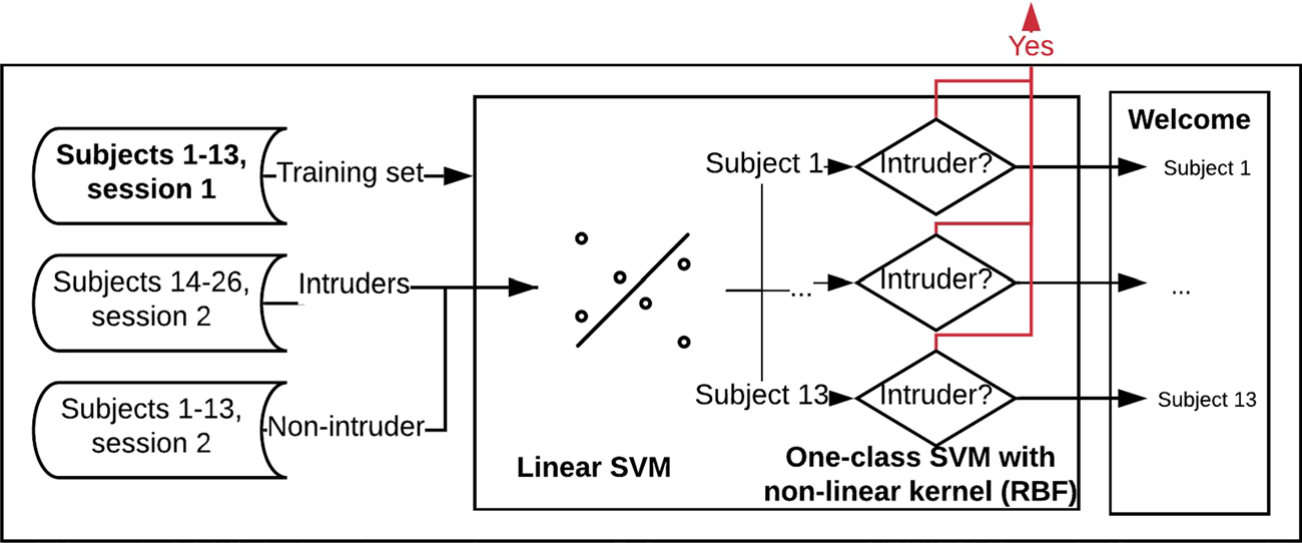
Flowchart of the second approach for intruder detection and subject identification.

Once the non-intruders and intruders subsets were defined, the signals were pre-processed and the features extracted. They can be used as input to the authentication system, which can be distributed in two different ways, as presented in Figs. 1 and 7. As mentioned previously, here, we used only the approach presented in Fig. 1. However, the use of a more complex system is required to fit certain important parameters and select the most relevant EEG channels, which in this case was analyzed as an optimization problem.

The problem to be optimized is defined by four unconstrained objectives: decrease the number of EEG channels, maximize the accuracy of the multi-class classification, maximize the number of accepted subjects with access, and maximize the number of intruders rejected. Each population size in each iteration is defined as 30, which was selected experimentally. The termination criterion for the optimization process is defined by the objective space tolerance, which is defined as 0.0001. This criterion is calculated every 10*th* generations. If optimization is not achieved, the process stops after a maximum of 500 generations.

**Figure 8:**
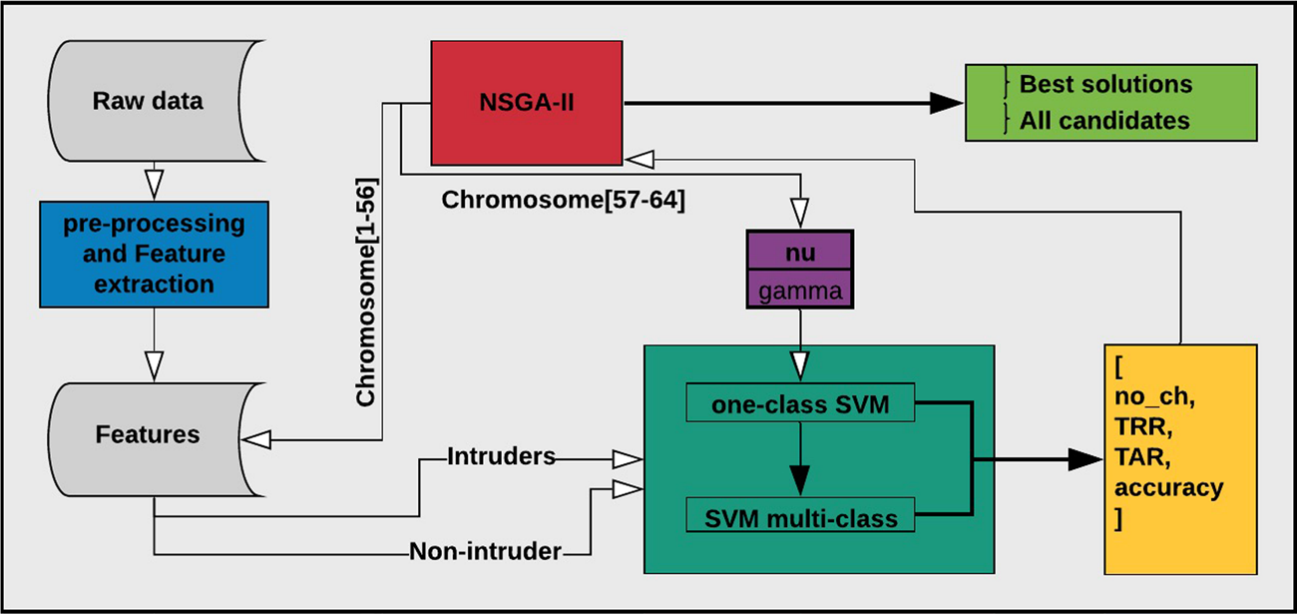
Example of the complete process for EEG channel selection using NSGA-II.

The chromosome created to represent the search space in the scalp is presented in Fig. 5, where genes 1–56 represent the EEG channels and the *nu* parameter is calculated using genes 57–60 and the *gamma* parameter calculated using genes 61–64. When calculating the *nu* and *gamma* parameters, the binary representation is converted to a decimal, which represents the position in a vector with the possible values for the parameter. Thus possible values were defined experimentally, which in a key-value array are {0: 0.000001, 1: 0.0001, 2: 0.0005, 3: 0.001, 4: 0.005, 5: 0.01, 6: 0.1, 7: 0.2, 8: 0.3, 9: 0.4, 10: 0.5, 11: 0.6, 12: 0.7, 13: 0.8, 14: 0.9, 15: 1.0}, for both *nu* and *gamma*. The complete process is illustrated in Fig. 8.

We extracted eight features per EEG channel from all subjects and for each instance following the previously explained method, in which the results are organized and stored for iterative use, as shown in Fig. 8. The entire process is then handled by NSGA-II or NSGA-III, which starts creating all possible candidates using a binary chromosome representation, for which the corresponding subset of features for the channels is obtained, represented as 1 for genes 1–56 of the chromosome, the *nu* parameter calculated using genes 57–60, and the *gamma* parameter calculated using genes 61–64.

Then, the obtained classification accuracy, number of accepted subjects with access, number of rejected subjects, and number of EEG channels used are returned to NSGA-II or NSGA-III to evaluate each chromosome in the current population. The process is repeated, creating different populations by the NSGA until the termination criterion is reached.

## Data Availability

The dataset used for this study can be found at BCI Challenge @ NER 2015.
